# The gut microbial composition in polycystic ovary syndrome with hyperandrogenemia and its association with steroid hormones

**DOI:** 10.3389/fcell.2024.1384233

**Published:** 2024-05-30

**Authors:** Miao Li, Qiurong Chang, Ye Luo, Jiaping Pan, Ye Hu, Binya Liu, Mengmeng Ma, Qiaoling Wang, Yi Guo, Qian Wang

**Affiliations:** ^1^ Center for Reproductive Medicine, Shanghai First Maternity and Infant Hospital, School of Medicine, Tongji University, Shanghai, China; ^2^ Department of Research and Education, Shanghai First Maternity and Infant Hospital, School of Medicine, Tongji University, Shanghai, China

**Keywords:** PCOS, microbiota, hyperandrogenemia, serum hormones, metabolism

## Abstract

**Background:** Polycystic ovary syndrome (PCOS) is characterized by excess androgens, ovulatory dysfunction, and polycystic ovaries. The mechanisms underlying ovulatory and metabolic disorders in PCOS remain elusive, hampering therapeutic development. Enhanced metabolic health correlates with increased microbiota gene content and microbial diversity. We aimed to explore the impact of gut microbiota and serum steroids on PCOS regulation associated with androgen excess.

**Methods:** The fecal samples of patients with hyperandrogenic PCOS (*n* = 14) and control group with PCOS (*n* = 14) were analyzed by 16S rRNA gene sequencing. The peripheral venous blood of all subjects was collected to detect serum hormones. The association between gut microbiota and serum hormones was analyzed with the R language.

**Results:** Our findings reveal that the hyperandrogenic PCOS group exhibits lower richness and diversity of gut microbiota compared to the control group. Characteristic genera in PCOS patients with hyperandrogenism include *Bifidobacterium, Enterobacteriaceae_unclassified, Streptococcus, Saccharimonadaceae, Enterococcus*, and *Eubacterium_nodatum_group*. Five hormones, including 5β-androsterone, deoxycorticosterone, corticosterone, 11-dehydrocorticosterone, and cortexolone, emerge as potential serum biomarkers for identifying patients with hyperandrogenic-PCOS (HA-PCOS). Furthermore, a lower vitamin D3 level may act as a susceptibility factor, suggesting that vitamin D3 supplementation could serve as a potential intervention for PCOS with hyperandrogenism.

**Conclusion:** Specific fecal microbiota and serum steroids may be used as characteristic markers for clinical diagnosis of hyperandrogenic-PCOS. This research enhances our understanding of the intricate interplay among hormones, gut microbiota, and hyperandrogenemia in patients with PCOS.

## Introduction

Polycystic ovary syndrome (PCOS) stands out as one of the most prevalent reproductive endocrine metabolic disorders, affecting an astounding 66 million women worldwide. Since 1990, its prevalence has surged by an alarming 30.4%, yet its etiology remains elusive (Bozdag et al., 2016; Safiri et al., 2022). PCOS is characterized by persistent menstrual irregularities, clinical or biochemical hyperandrogenism, and polycystic ovarian morphology. This complex disorder is linked to various metabolic derangements, including obesity, insulin resistance (IR), and type 2 diabetes (T2D) (Joham et al., 2022). Despite numerous expert-based diagnostic criteria, a universally accepted standard is still lacking. PCOS presents with a highly heterogeneous clinical profile, manifesting a spectrum of symptoms with diverse consequences for affected women (Copp et al., 2021). Therefore, it is imperative to discern the distinct features of PCOS subtypes and implement corresponding interventions.

As mentioned earlier, hyperandrogenism is a hallmark feature of PCOS. In particular, 80%–90% of women with oligomenorrhea exhibit elevated androgen levels. The hyperandrogenic subtype (HA-PCOS) is characterized by relatively high levels of testosterone (T) and dehydroepiandrosterone-sulfate, along with mild metabolic disorders, encompassing approximately 25% of patients with PCOS. Hyperandrogenemia may contribute to reproductive disorders, IR, and metabolic imbalances, such as glucose and lipid metabolic disturbances (Giampaolino et al., 2021). Furthermore, androgen excess may lead to various chronic metabolic diseases, including obesity, non-alcoholic fatty liver disease (NAFLD), T2D, hypertension, and dyslipidemia.

The gut microbiota, deemed an indispensable “microbial organ” of the human body, profoundly influences host material metabolism and immune function, playing a pivotal role in endocrine and metabolic diseases (Sonnenburg and Bäckhed, 2016; Guo et al., 2022), including reproductive and gynecological diseases (Chen et al., 2020; Graham et al., 2021). PCOS incidence is closely intertwined with gut microbiota (Torres et al., 2018). Notably, the richness and diversity of gut microbiota in patients with PCOS are lower than those in healthy women (Zhou et al., 2020). Compared with non-obese patients with PCOS, *Coprococcus*_2 and *Lactococcus* emerge as characteristic gut microbiota in obese patients with PCOS. These distinct bacterial genera likely influence the intestinal environment of the host via different enrichment and metabolism patterns, thereby impacting PCOS occurrence and development with and without obesity. A recent study by Qi et al., 2019 demonstrated that mice with fecal microbiota transplantation from patients with PCOS developed a PCOS-like syndrome, highlighting the causal role of gut microbiota in PCOS. This study further proposed that the modification of gut microbiota, coupled with bacteria-related bile acid and immune changes, may represent a novel treatment for PCOS. However, to date, no study has explored the association between gut microbiota and hyperandrogenism in patients with PCOS or identified potential landmark microbiota in patients with HA-PCOS.

In the present study, we characterized the gut microbiota and serum steroids in patients with PCOS with and without hyperandrogenism, delving into the correlations between gut microbiota and serum hormones. Our aim is to unveil the potential mechanism by which gut microbiota influences the development of hyperandrogenism in patients with PCOS from a new perspective.

## Results

### Clinical baseline characteristics of participants

All subjects are Chinese Han women from Shanghai First Maternity and Infant Hospital with similar eating habits. The clinical characteristics of all participants were summarized in Table 1. The group with the HA-PCOS subtype exhibited significantly higher T levels compared to the control PCOS group, with no significant age difference between the two groups (Table 1). Notably, the body mass index (BMI) in patients with HA-PCOS was significantly elevated compared to the control group (Table 1). Despite no statistical significance, an upward trend was observed in triglycerides (TG) and total cholesterol (TC) in the HA-PCOS group (Table 1). Sex hormones, including estradiol (E2) and progesterone (P), showed no significant differences between women with HA-PCOS and PCOS controls (Table 1).

**TABLE 1:** Clinical baseline characteristics of participants.

Parameters	Control PCOS (*n* = 14)	HA-PCOS (*n* = 14)
Age (years)	29.00 ± 2.13	30.75 ± 3.19
BMI (kg/m2)	22.53 ± 2.40	24.16 ± 2.86**
T (μg/L)	0.23 ± 0.66	0.50 ± 0.11**
E2 (μg/L)	0.56 ± 1.21	0.50 ± 0.47
P (μg/L)	0.29 ± 0.34	0.33 ± 0.68
TG (mmol/L)	1.22 ± 0.38	1.38 ± 0.58
TC (mmol/L)	4.44 ± 0.68	4.67 ± 0.68

BMI, body mass index; T, testosterone; E2, estradiol; P, progesterone; TG, triglycerides; TC, total cholesterol; PCOS, polycystic ovary syndrome. Data are presented as mean ± standard deviation. The difference between control PCOS and HA-PCOS, was compared using the independent-sample *t*-test approach, and *p* < 0.05 was considered statistically significant. *p < 0.05; **p < 0.01.

### Gut microbiota diversity and community composition in patients with HA-PCOS

**FIGURE 1:**
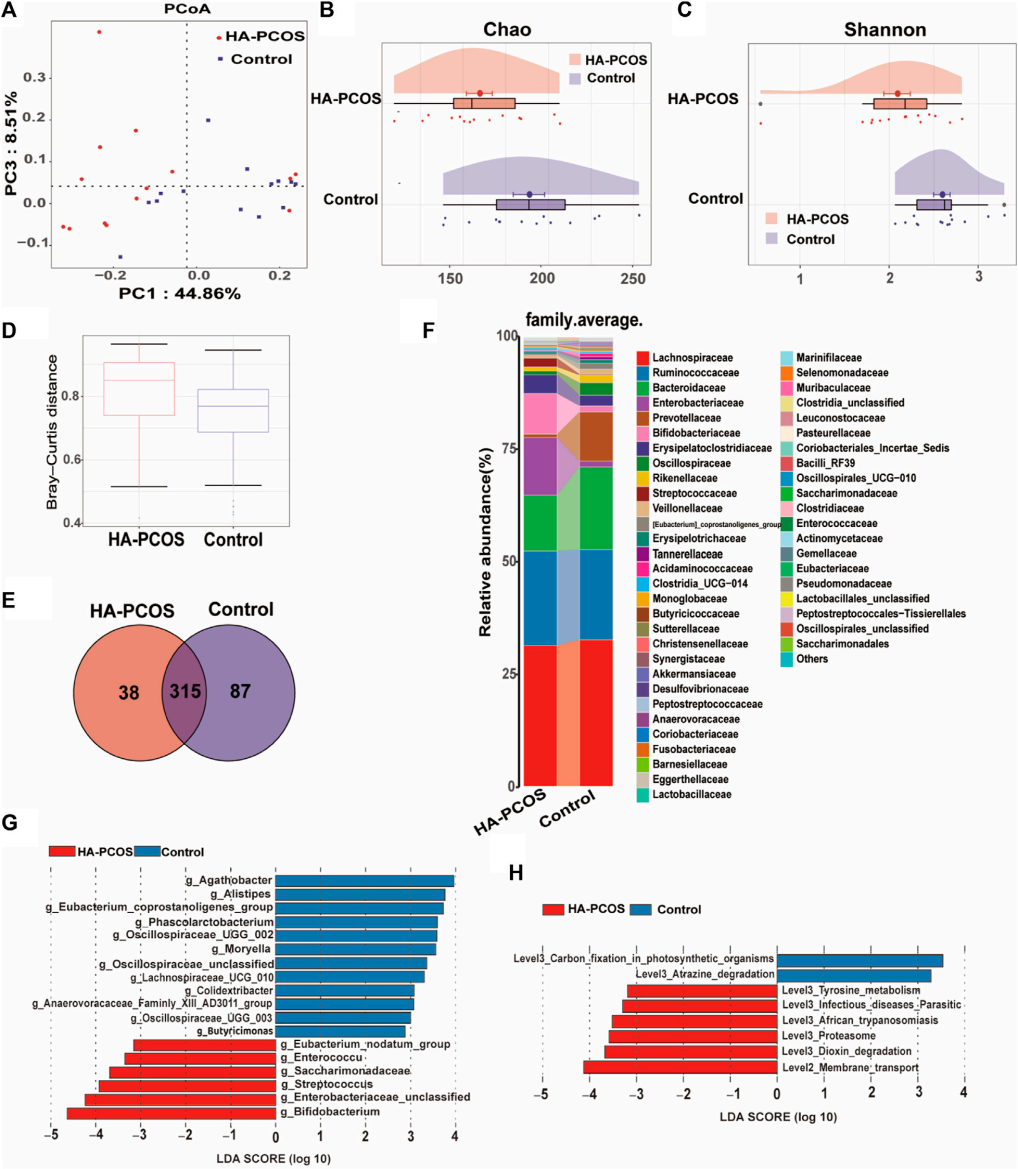
Gut microbial alterations in women with HA-PCOS compared to the control PCOS group **(A)**. PCoA was performed based on the weighted UniFrac distance. The distance of each coordinate point represents the degree of aggregation and dispersion between samples. **(B)**. The Chao 1 estimator illustrates the community richness of alpha-diversity. Differences in Chao1 between the two groups were compared using the two-tailed Mann–Whitney U-test. Data are shown as mean ± standard deviation. **(C)**. The Shannon index illustrates the community diversity of alpha-diversity. Differences in the Shannon index were compared using the two-tailed Mann–Whitney U-test. Data are shown as mean ± standard deviation. **(D).** The beta diversity (Bray–Curtis distance) illustrates the control and PCOS groups at the gene level. For box plots, the midline represents the median, and the box represents the interquartile range between the first and third quartiles. **(E)**. The Venn diagram displays the numbers of shared and unique OTUs between the two groups. Different colors represent different sample groups. **(F)**. The heatmap shows the relative abundance of gut microbiota in the two groups at the family level. **(G)**. Bar chart displays the distribution of linear discriminant analysis (LDA) values, showing biomarkers with LDA scores above a certain threshold that exhibit statistically significant differences. The length of the bars represents the magnitude of the impact of significantly different species. The LDA effect size (LEfSe) approach was used to identify the discriminatory microbial genera with the LDA score (log 10) ≥ 2.0 and the adjusted *p* < 0.05. Blue bars represent microbial genera significantly enriched in the control group, whereas red bars represent microbial genera significantly enriched in the HA-PCOS group. **(H)**. The LDA analysis results and LDA bar chart of the differentially represented metabolic pathways selected in the KEGG pathway using the LEfSe method between the groups are shown.

To discern distinctive expression patterns in patients with HA-PCOS, gut microbiome analysis was conducted on fecal samples. The Shannon–Wiener curve was employed to determine if the sequencing quantity adequately estimated species richness (Supplementary Figure S1A). The curve displayed a seemingly flattened trend, suggesting sufficient sequencing depth to reflect species diversity in the samples (Supplementary Figure S1A). Principal coordinate analysis (PCoA) illustrated a distinct clustering pattern between samples from patients with HA-PCOS and controls (Figure 1A). Alpha diversity analysis revealed that the Chao1 index, reflecting community richness, and the Shannon index, representing community diversity, were significantly decreased in the HA-PCOS group compared to the control group (*p* < 0.01, Figures 1B,C). Importantly, beta diversity, calculated by Bray–Curtis distance of HA-PCOS microbiomes, was significantly increased compared to that of control patients with PCOS, indicating a more homogeneous community structure among individuals in the control group (Figure 1D). Operational taxonomic units (OTUs), defined as groups of sequences sharing 97% identity, serve as a fundamental unit for species annotation, community diversity, and group differences. The Venn diagram visually displayed the distribution of OTU numbers in the two groups, highlighting common and unique characteristics. Analyses revealed that 315 OTUs were present in both HA-PCOS and control groups, with 38 unique to HA-PCOS and 87 unique to control patients (Figure 1E). The histogram depicted the top 50 bacteria families in abundance in the two groups (Figure 1F). Overall, the relative abundance of Bacteroidaceae, Prevotellaceae, and Oscillospiraceae was reduced in the HA-PCOS group, known for their roles in digestion and maintaining healthy metabolism (Han et al., 2023; He et al., 2023). Conversely, Enterobacteriaceae, Bifidobacteriaceae, and Streptococcaceae were more concentrated in patients with HA-PCOS (Figure 1F).

We further characterized the gut microbiota composition biomarkers at the genus level. In this study, a total of 164 strains were obtained from all fecal samples. The linear discriminant analysis (LDA) effect size (LEfSe) approach was employed to identify differential microbial genera between the HA-PCOS and control groups. As depicted in Figure 1G, 18 strains significantly differed between HA-PCOS and control groups. Among them, six species were notably elevated in HA-PCOS, including *Bifidobacterium, Enterobacteriaceae_unclassified, Streptococcus, Saccharimonadaceae, Enterococcus*, *and Eubacterium_nodatum_group* (Figure 1G). Consistent with Figure 1F, *Streptococcus* belongs to the Streptococcaceae family, and members from the Enterobacteriaceae family were also identified. These genera were associated with pathways related to infectious diseases, parasitic diseases, African trypanosomiasis, or other pathogenesis, which might contribute to metabolic disorders or inflammation in patients with HA-PCOS (Figure 1H). Conversely, the remaining 12 were depleted in HA-PCOS, such as *Agathobacter*, *Alistipes*, *Eubacterium_coprostanoligenes_group*, *Phascolarctobacterium*, *Oscillospiraceae_UCG_002*, *Moryella*, *Oscillospiraceae_unclassified*, *Lachnospiraceae_UCG_010*, *Colidextribacter*, *Anaerovoracaceae_Family_XIII_AD3011_group*, *Oscillospiraceae_UCG_003*, and *Butyricimonas* (Figure 1G). Most of these genera are known for their beneficial roles in digestion and absorption of dietary fats, potentially improving metabolic disorders including obesity or T2D (Bubeck et al., 2023; Zhang, Wang, et al., 2023).

### Identification of characteristic serum steroids in patients with PCOS

**FIGURE 2:**
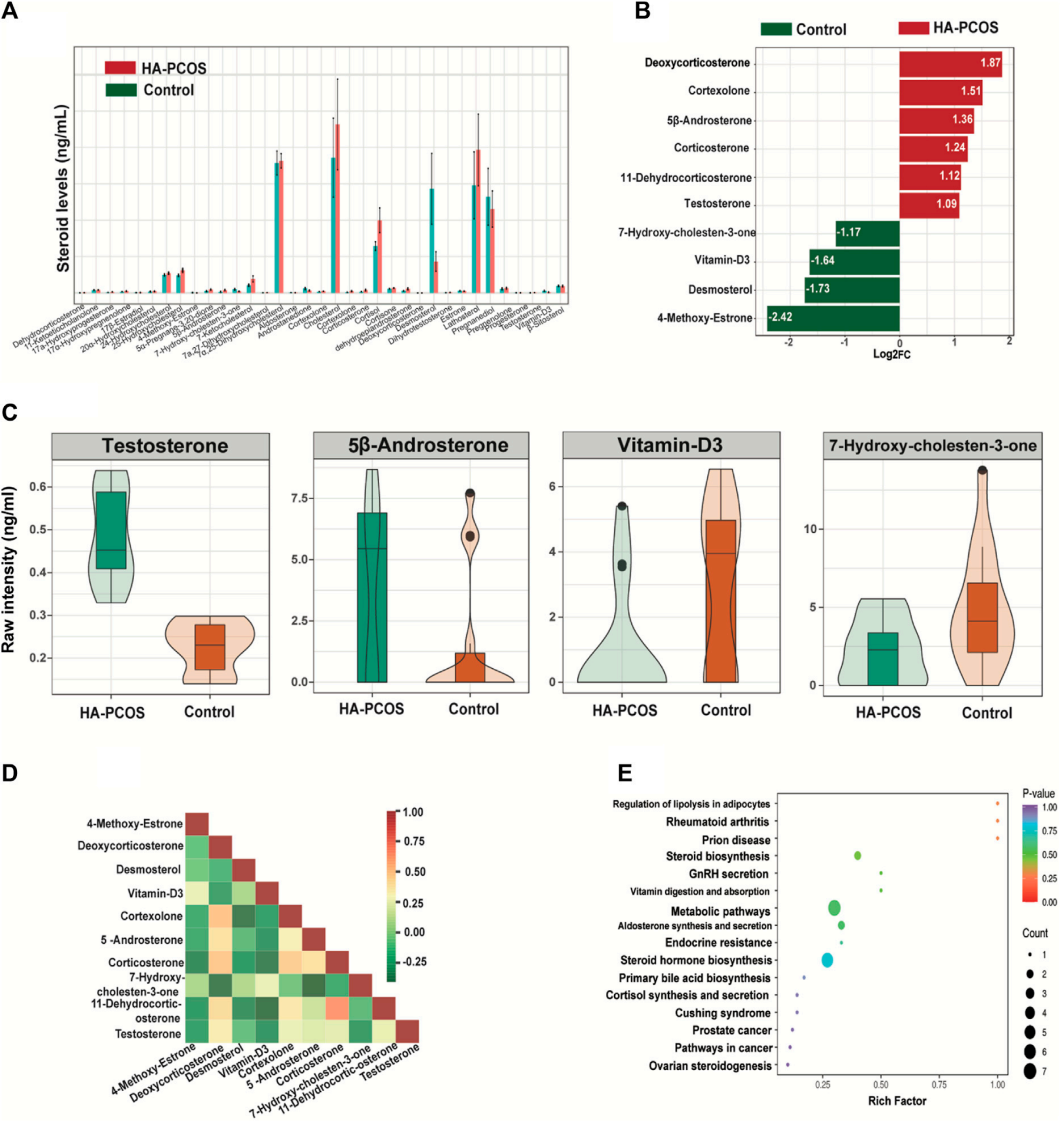
Serum steroid hormones in women with HA-PCOS compared to the control PCOS group **(A)**. The column chart shows the abundance of steroids in the two groups. Data are depicted as mean ± standard deviation. **(B)**. The significantly differential metabolites between the HA-PCOS and control groups. The *X*-axis represents the log2 fold change of the differential metabolites. The *Y*-axis indicates the differential metabolites. Red bars represent upregulated metabolites in the HA-PCOS group, and green bars indicate downregulated metabolites in the control group. **(C)**. The violin plot shows the relative levels of testosterone, 5β-androsterone, vitamin D3, and 7-hydroxy-cholesten-3-one in the two groups, respectively. A violin plot is a combination of a box plot and a density plot used to display the distribution of data and its probability density. The central box represents the interquartile range, and the thin black lines extending from it indicate the 95% confidence interval. **(D)**. Pearson’s correlation analysis was performed on the significantly differential metabolites obtained via the selection criteria. Different colors represent the strength of the Pearson’s correlation coefficient; red indicates a strongly positive correlation, green indicates a strongly negative correlation, and the darker the color, the larger the absolute value of the correlation coefficient between samples. **(E)**. KEGG pathway enrichment analysis was conducted according to the differential metabolite results. The rich factor is the ratio of the number of differential metabolites to the total number of metabolites annotated in a pathway. A *p*-value closer to 0 indicates a more significant enrichment.

Steroid levels, including progestogen, vitamin, androgen, cortical hormone, estrogen, and sterols, were further assessed in patients with HA-PCOS and control PCOS (Supplementary Table). The coefficient of variation values reflecting the degree of data dispersion, indicated the reliability of the detection methods (Supplementary Figure S2A). Principal component analysis (PCA) demonstrated distinct clustering patterns between samples from patients with HA-PCOS and controls (Supplementary Figure S2B). The relative abundance of serum steroids in patients with HA-PCOS and the control group were showed in Figure 2A. Moreover, 10 hormones showed significant changes in HA-PCOS (Figure 2B). Alongside T, deoxycorticosterone, cortexolone, 5β-androsterone, corticosterone, and 11-dehydrocorticosterone were upregulated in the HA-PCOS group (Figures 2B,C). Derived from deoxycorticosterone, 11-dehydrocorticosterone is a steroid hormone serving as an intermediate in the synthesis of corticosterone and aldosterone, playing a key role in regulating electrolyte balance. Notably, a positive association was revealed between 11-dehydrocorticosterone and corticosterone through correlation analysis (Figure 2D).

In contrast, 4-methoxy-estrone, desmosterol, vitamin-D3, and 7-hydroxy-cholesten-3-one were downregulated in patients with HA-PCOS (Figures 2B,C), with T showing a negative correlation with these hormones (Figure 2D). Subsequently, Kyoto Encyclopedia of Genes and Genomes (KEGG) pathway analysis on these 10 differential hormones indicated their involvement in metabolic pathways and steroid hormone biosynthesis (Figure 2E). Analyzing these hormones could be crucial for managing patients with HA-PCOS.

### Correlation analysis of differential fecal gut microbiota and serum steroids hormone in the HA-PCOS group

**FIGURE 3:**
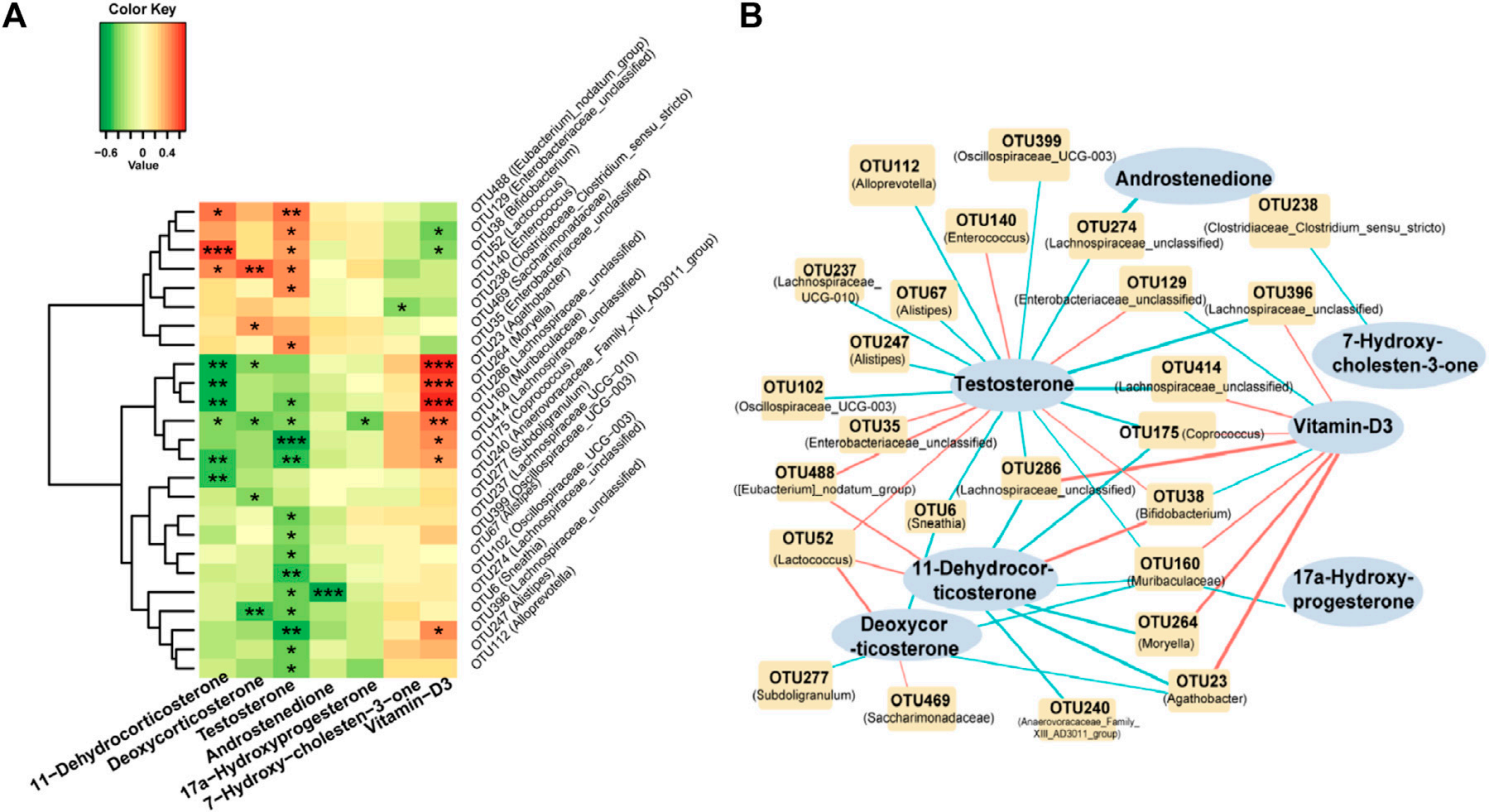
Correlation analysis of gut microbiota and serum steroid hormones in the HA-PCOS group **(A)**. Correlation between seven serum metabolites and the top 25 differential fecal gut microbiota in the HA-PCOS group. The gradient colors in each square correspond to the correlation coefficients between the respective species and metabolites, where red indicates a positive correlation and green indicates a negative correlation. **(B)**. The correlation network connects the correlated gut microbiota and steroid hormones. Red links indicate positive correlations, whereas blue links represent negative correlations. The thickness of the links corresponds to the absolute value of the correlation coefficient.

We conducted a comprehensive analysis of the correlation between seven crucial differential metabolites and the top 25 genera in the HA-PCOS group (Figure 3A). Testosterone, the most significantly upregulated sex hormone in the HA-PCOS group, exhibited intricate interactive relationships with multiple microbial communities (Figure 3A). Notably, the abundance of testosterone negatively correlated with *Lachnospiraceae_unclassified* (*p* < 0.001) and 12 other OTUs, whereas it positively correlated with six OTUs, including *Eubacterium_nodatum_group* (*p* < 0.01) (Figure 3A). Furthermore, the level of vitamin D3 exhibited a positive correlation with *Agathobacter*, *Moryella*, and *Lachnospiraceae_unclassified* (*p* < 0.001), while showing a negative correlation with *Enterobacteriaceae_unclassified* (*p* < 0.05). This suggests that the relative deficiency of vitamin D3 in patients with HA-PCOS might be attributed to flora disequilibrium. Additionally, the level of 11−dehydrocorticosterone positively correlated with the abundance of *Bifidobacterium* (*p* < 0.001) and *Lactococcus* (*p* < 0.05), but negatively correlated with the abundance of *Agathobacter*, *Moryella*, and *Lachnospiraceae_unclassified* (*p* < 0.01) (Figure 3A). On the other hand, androstenedione, 17α−hydroxyprogesterone, and 7−hydroxy−cholesten−3−one appeared to have minimal relationships with the gut microbiota (Figure 3A). To visually represent the relationship between gut microbiota and serum hormones, a correlation diagram was showed in Figure 3B.

## Materials and methods

### Participants

Between June 2020 and June 2021, a total of 28 patients diagnosed with PCOS were recruited from the Shanghai First Maternity and Infant Hospital. Inclusion criteria adhered to the PCOS diagnostic criteria revised by the Rotterdam conference in the Netherlands in 2003 including oligoovulation and/or anovulation, hyperandrogenism, and polycystic ovarian morphology by ultrasound. Exclusion criteria comprised the other causes of hyperandrogenism such as hyperprolactinemia, Cushing syndrome, and nonclassical congenital adrenal hyperplasia; use of oral contraceptives, antiandrogens, or insulin sensitizers within 3 months prior to recruitment; pregnancy; any mental or organic diseases; use of corticosteroids or sex steroids; drug use and alcohol abuse in past 2 years; and the use of antibiotics, probiotics, or prebiotics in the past 3 months. The participants were categorized into the HA-PCOS (*n* = 14) and control PCOS (*n* = 14) groups based on serum T levels. The study received approval from the Ethics Committee of Shanghai First Maternity and Infant Hospital (NO. KS20207) and informed consent was obtained from each participant before the study.

### Collection of peripheral venous blood and fecal samples

On the third day of the menstrual cycle, peripheral venous blood was collected from participants, and levels of sex hormones (E2, T, and P) were immediately detected by the clinical laboratory of our hospital using chemiluminescent immunoassay. Fecal samples were obtained from participants 3–5 days following menstruation. Participants were instructed to follow a carbohydrate-based diet (300 g/day) for 3 days before sampling. Standardized procedures were employed for sample collection and treatment to minimize the effects of non-sample factors on gut flora and metabolites. Fresh fecal samples (10 g each) were collected from each participant using a sterile plastic spoon and tube. Samples were placed in an icebox, transported to the laboratory within 2 h, and stored at −80°C. The structural characteristics of gut microbiota in the two groups were analyzed using 16S rRNA gene sequencing.

### Detection and analyze of gut microbiota in patients

DNA extraction, polymerase chain reaction (PCR) amplification, and sequencing: All samples underwent standardized procedures for DNA extraction and PCR amplification conducted by the same laboratory personnel. Samples were suspended in 790 μL of sterile lysis buffer (4 M guanidine thiocyanate; 10% N-lauroyl sarcosine; 5% N-lauroyl sarcosine-0.1 M phosphate buffer [pH 8.0]) in a 2 mL screw-cap tube containing 1 g glass beads (0.1 mm BioSpec Products, Inc., United States). The mixture was vigorously vortexed and incubated at 70°C for 1 h. Following bead beating for 10 min at maximum speed, DNA extraction was performed using The E.Z.N.A.^®^ Stool DNA Kit (Omega Bio-tek, Inc., GA) according to the manufacturer’s instructions, with the exception of lysis steps, and stored at −20°C for further analysis.

The extracted DNA from each sample served as the template for amplifying the V3-V4 region of 16S rRNA genes. The primers (341F: 5′- CCTACGGGNGGCWGCAG -3′ and 805R: 5′-GACTACHVGGGTATCTAATCC-3′) targeting positions 341 to 805 in the *Escherichia coli* 16S rRNA gene were employed for PCR amplification. PCR reactions were conducted in an EasyCycler 96 PCR system (Analytik Jena Corp., AG). Subsequently, products from different samples were indexed, mixed at equal ratios, and sequenced by Shanghai Mobio Biomedical Technology Co. Ltd. using the Miseq platform (Illumina Inc., United States) following the manufacturer’s instructions.

Bioinformatics analyses: Clean data was extracted from raw data using USEARCH (version 11.0.667) with the following criteria: 1) Each index with zero mismatch was used to extract sequences of each sample. 2) Sequences with overlapping of <16 bp were discarded. 3) The error rate of the overlapping >0.1 was discarded. 4) Sequences less than 400 bp after merging were discarded. Quality-filtered sequences were clustered into unique sequences using UPARSE, and singletons were omitted in this step. OTUs were classified based on 97% similarity, and chimeric sequences were removed using UPARSE (version 7.1 http://drive5.com/uparse/). Taxonomic annotations were performed using the SILVA reference database (SSU138) in qiime2-2020.11. Alpha diversity metrics (Chao 1 estimator, Shannon–Wiener diversity index, and Shannon diversity index) were assessed using Mothur v1.42.1. The nonparametric Mann–Whitney U test (R 3.6.0 package stats) was employed for pairwise comparisons. Bray–Curtis was calculated in QIIME (v1.9.1). PCoA plots were generated in R (version 3.6.0) using the vegan 2.5-7 package. LEfSe was used to detect taxa with differential abundance among groups (LEfSe 1.1, https://github.com/SegataLab/lefse). Functional abundances were predicted using PICRUSt2 v2.4.1 (https://github.com/picrust/picrust2/wiki) based on 16S rRNA gene sequences.

Raw sequencing data of the 16S rRNA gene V3–V4 regions and accompanying information are available in the Sequence Read Archive database under accession number PRJNA1058122.

### Detection of serum steroids using targeted metabolomics

After the sample was thawed, the sample was vortexed for 10 s. 100 μL of the sample was transferred to a centrifuge tube and mixed with 400 μL of methanol. 10 μL internal standard was added into the extract as internal standards (IS) for the quantification, vortexed for 10 min, stand on ice for 10 min and centrifuged at 12,000 r/min for 5 min at 4°C. Take 400 μL of supernatant into a new centrifuge tube and concentrate it at 20°C until it is completely dry. Then the sample was redissolved with 100 μL methanol, vortex for 5 min, centrifuged at 12,000 r/min for 3 min at 4°C. After centrifugation, 80 μL of the supernatant was transferred for further LC-MS analysis.

UPLC Conditions: The sample extracts were analyzed using an LC-ESI-MS/MS system (UPLC, ExionLC AD, https://sciex.com.cn/; MS, QTRAP^®^ 6500+ System, https://sciex.com/). The analytical conditions were as follows, HPLC: column, Phenomenex Kinetex C18 (1.7 µm, 100 mm × 2.1 mm i.d.); solvent system, 30% acetonitrile/water with 0.04% Acetic acid (A), 50% acetonitrile/isopropanol with 0.04% Acetic acid (B); The gradient was started at 5% B (0 min–1.0 min), increased to 90% B (1.0–10 min), 90% B (10–12.5 min), finally ramped back to 5% B (12.6–15 min); flow rate, 0.35 mL/min; temperature, 40°C; injection volume: 5 μL.

ESI-MS/MS Conditions: AB 6500+ QTRAP^®^ LC-MS/MS System, equipped with an ESI Turbo Ion-Spray interface, was controlled by Analyst 1.6 software (AB Sciex). The ESI source operation parameters were as follows: ion source, turbo spray; source temperature 550°C; ion spray voltage (IS) 5500 V (Positive); curtain gas (CUR) were set at 35.0 psi; DP and CE for individual MRM transitions was done with further DP and CE optimization. A specific set of MRM transitions were monitored for each period according to the neurotransmitters eluted within this period.

Qualitative and quantitative principles of metabolites: Metabolites were quantified by multiple reaction monitoring (MRM) using triple quadrupole mass spectrometry. In MRM mode, the first quadrupole screened the precursor ions for the target substance and excluded ions of other molecular weights. After ionization induced by the impact chamber, the precursor ions were fragmented, and a characteristic fragment ion was selected through the third quadrupole to exclude the interference of non-target ions. After obtaining the metabolite spectrum data from different samples, the peak area was calculated on the mass spectrum peaks of all substances and analyzed by standard curves.

Unsupervised PCA was performed by statistics function prompt within R (www.r-project.org).

Significantly regulated metabolites between groups were determined by absolute Log_2_fold change.

Identified metabolites were annotated using KEGG compound database (http://www.kegg.jp/kegg/compound/), annotated metabolites were then mapped to KEGG Pathway database (http://www.kegg.jp/kegg/pathway.html).

### Correlation analysis of differential fecal gut microbiota and serum steroids

Differential bacteria and hormones between HA-PCOS and control PCOS group were identified and then calculate the Spearman correlation coefficient. The correlation was significant when the *p*-value was <0.05. The correlation network was drawn by Cytoscape Software.

## Discussion

In this study, we investigated the differences in gut microbiota and serum hormone levels and explored their correlation in PCOS patients with or without hyperandrogenism. The HA-PCOS group exhibited a higher incidence of dyslipidemia and NAFLD compared to the control PCOS patients, indicating a stronger association between hyperandrogenic features and severe lipid metabolic dysfunction. Previous studies have also reported that elevated maternal testosterone levels are linked to adverse pregnancy outcomes such as low birth weight and an increased risk of preterm delivery, accompanied by fetal membrane rupture, cervical dilatation, and live delivery (Mecenas et al., 1996; Zheng et al., 2022).

Combining alpha and beta diversity analyses, we observed dysbiosis in the intestines of hyperandrogenic patients with PCOS. 18 differential bacterial strains were found between the HA-PCOS and control PCOS groups. Six species, including *Bifidobacterium*, were significantly elevated in the HA-PCOS group. *Bifidobacterium* is known to play a beneficial role in maintaining gut health and overall wellbeing (Van Syoc et al., 2023). The ecological role of *Saccharimonadaceae* bacteria is not fully understood (Zhang, Meng, et al., 2022), but they are believed to contribute to carbon and nutrient cycling in their environment (Zhang, Meng, et al., 2022). Genera such as *Enterobacteriaceae, Streptococcus*, and *Enterococcus*, which elevated in HA-PCOS, are generally considered pro-inflammatory or opportunistic pathogens (Chen et al., 2023; Taylor et al., 2023; Zhang, Tan, et al., 2023). The remaining 12 genera were downregulated in HA-PCOS. *Alistipes* (From Bacteroidaceae family), *Oscillospiraceae* (from Oscillospiraceae family), *Lachnospiraceae*, and *Butyricimonas* play a crucial role in breaking down and fermenting complex dietary fibers, producing short-chain fatty acids (SCFAs) as byproducts. SCFAs are associated with various health benefits (Pisaniello et al., 2023; Van den Abbeele et al., 2022; Wang et al., 2023). A recent study revealed SCFA absorption was decreased in PCOS rats (Lin et al., 2021). SCFAs are critical for maintaining glucose and insulin homeostasis and mitigating chronic inflammation in the body (Kim, Park, and Kim, 2014). These genera may contribute to increasing the levels of TG and TC in patients with HA-PCOS. *Agathobacter* was associated with the degradation of organic matter because they exhibit certain enzymatic activities involved in the breakdown of complex compounds (Zhang, Wang, et al., 2023). *Eubacterium_coprostanoligenes* has been linked to the metabolism of bile acids and cholesterol, along with benefits for gut health (Bubeck et al., 2023). The genus *Phascolarctobacterium* has been reported to metabolize complex dietary fibers and produce volatile fatty acids (He et al., 2023). *Moryella* bacteria are believed to contribute to the production of essential nutrients, such as vitamin B and amino acids, which are beneficial to the host animal. They are also involved in the maintenance of a stable microbial gut ecosystem (Li et al., 2020).

The serum metabolomics analyses revealed inhibitions in 4-methoxy-estrone, desmosterol, vitamin-D3, and 7-hydroxy-cholesten-3-one in patients with HA-PCOS, with particular emphasis on the potential significance of vitamin D3. Vitamin D3, known for its role in calcium absorption and bone mineralization, has been associated with PCOS, where vitamin D3 deficiency is common and linked to higher androgen levels and IR (Menichini and Facchinetti 2020). Although the precise mechanisms by which vitamin D3 influences hyperandrogenism are still being elucidated, optimizing its levels may hold promise as a therapy for PCOS (Lejman-Larysz et al., 2023; Joham et al., 2022). Our study corroborates the reduced levels of vitamin D3 in patients with HA-PCOS, providing further support for its involvement in androgen metabolism. Desmosterol is a precursor of cholesterol and an intermediate in the cholesterol biosynthesis pathway, whereas 7-hydroxy-cholesten-3-one is a cholesterol derivative. Both of them contribute to lipid metabolism and are important in maintaining cellular function (Camilleri and BouSaba 2023). Elevated levels of these metabolites in patients with HA-PCOS underscore disruptions in lipid metabolism, which might contribute to the higher BMI in these patients. Additionally, six hormones, including corticosterone, 5β-androsterone, and four cortical hormones, were increased in patients with HA-PCOS. Corticosterone, a stress-responsive steroid hormone, influences metabolism and immune function (Soma et al., 2023). 5β-androsterone, a testosterone metabolite, contributes to androgenic activity, albeit less potent than other androgens (Iannone et al., 2021). The four cortical hormones exhibit aldosterone-like actions, with excess levels linking to adverse health effects such as weight gain, high blood pressure, osteoporosis, and impaired immune function, consistent with PCOS phenotypes (Nakao et al., 2023; Jimeno and Verhulst 2023; Morgan et al., 2022; Kainuma et al., 2009).

In the present study, we found that serum T level negatively correlated with the abundance of *Lachnospiraceae_unclassified, Coprococcus, Muribaculaceae,* and *Oscillospiraceae,* whereas alterations in the abundance and diversity of these florae have been observed in inflammatory bowel disease, obesity, T2D and other metabolic disorders (Kim et al., 2022). Moreover, they could ferment dietary fibers and produce SCFAs, which are critically important for maintaining glucose and insulin homeostasis and ameliorating chronic inflammation throughout the body (Zhang et al., 2022). In turn, the abnormal glucose metabolism and compensatory hyperinsulinism contributed to hyperandrogenism, thereby disrupting the gut dysbiosis and metabolites.

In summary, our study unveiled characteristic changes in serum hormones and gut microbiota in hyperandrogenic patients with PCOS. Notably, six intestinal species, *Bifidobacterium, Enterobacteriaceae_unclassified, Streptococcus, Saccharimonadaceae, Enterococcus*, and *Eubacterium_nodatum_group*, emerged as characteristic markers in these patients. Additionally, five hormones, 5β-androsterone, deoxycorticosterone, corticosterone, 11-dehydrocorticosterone, and cortexolone, were identified as characteristic metabolites in patients with HA-PCOS. Moreover, the relative deficiency of vitamin D3 emerged as a noteworthy aspect for further exploration in understanding PCOS. These findings contribute to a deeper understanding of the intricate interplay between gut microbiota and serum hormones in hyperandrogenic PCOS, offering potential avenues for targeted interventions and personalized therapeutic approaches.

## Data Availability

The original contributions presented in the study are publicly available. This data can be found here: https://www.ncbi.nlm.nih.gov/bioproject/PRJNA1058122/

## Abbreviations

PCOS, Polycystic ovary syndrome; IR, insulin resistance; T2D, type 2 diabetes; HA-PCOS, hyperandrogenic-PCOS; T, testosterone; NAFLD, non-alcoholic fatty liver disease; BMI, body mass index; TG, triglycerides; TC, total cholesterol; E2, estradiol; P, progesterone; PCoA, Principal coordinate analysis; OTUs, Operational taxonomic units; LDA, The linear discriminant analysis; LEfSE, LDA effect size; PCA, Principal component analysis; PCR, polymerase chain reaction.
