# Supplementary material for: The gut microbial composition in polycystic ovary syndrome with hyperandrogenemia and its association with steroid hormones
Source: Front Cell Dev Biol. 2024 May 30;12:1384233. doi: 10.3389/fcell.2024.1384233 (PMC11169812; doi:10.3389/fcell.2024.1384233)
Supplement: Supplementary file 1 [file DataSheet1.docx]

**Supplemental Figures**

**
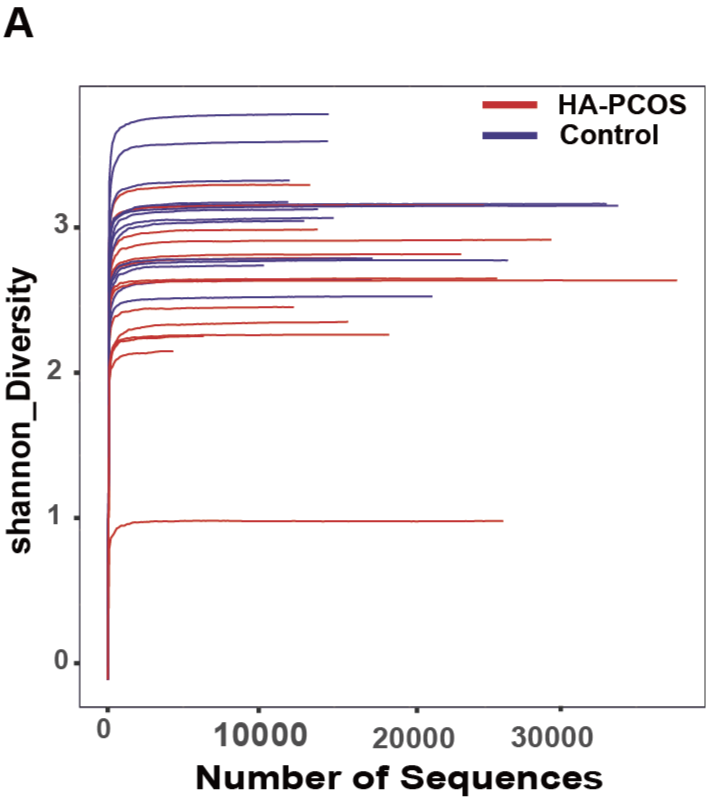
**

**Figure. S1 Gut microbial alterations in women with HA-PCOS compared to the control PCOS group**

A. The Shannon–Wiener index indicates that the sequencing data quantity is sufficiently large to capture a significant portion of the microbial information in the two samples.


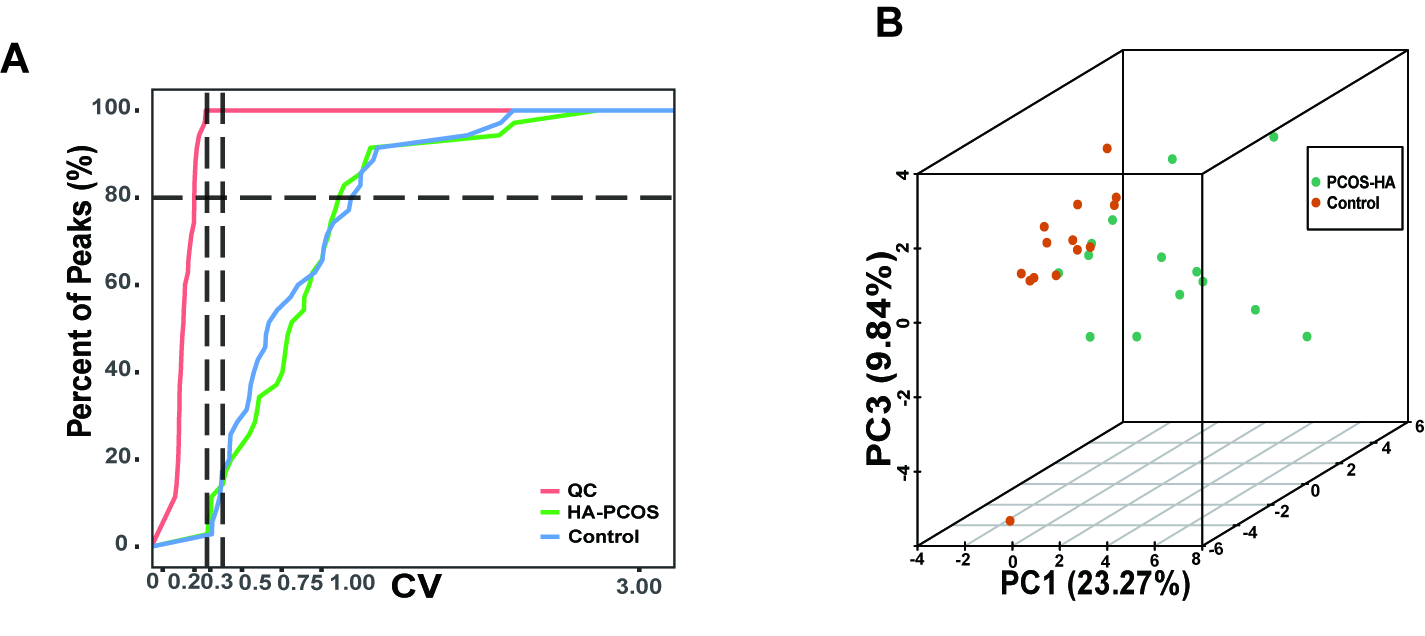


**Figure. S2 Serum steroid hormones in women with HA-PCOS compared to the control PCOS group**

**A.** The coefficient of variation is a measure of the dispersion and variability of data, calculated as the ratio of the standard deviation to the mean of the original data.

**B.** PCA shows a significantly different clustering pattern between the HA-PCOS and control groups.

**Supplemental table.**

**The list of 43 types of steroid hormones**

| Index | Class |
| --- | --- |
| 11-Hydroxyandrosterone | androgen |
| 11-Ketoetiocholanolone | androgen |
| Testosterone | androgen |
| Androstenedione | androgen |
| dehydroepiandrosterone | androgen |
| Dihydrotestosterone | androgen |
| Androstenedione | androgen |
| 5β-Androsterone | androgen |
| Androsterone | androgen |
| Aldosterone | cortical hormone |
| Cortisol | cortical hormone |
| Cortisone | cortical hormone |
| 11-Dehydrocorticosterone | cortical hormone |
| Corticosterone | cortical hormone |
| Cortexolone | cortical hormone |
| Deoxycorticosterone | cortical hormone |
| 16α-Hydroxy-Estrone | estrogen |
| 2-Hydroxy-Estrone | estrogen |
| 17β-Estradiol | estrogen |
| 17a-Estradiol | estrogen |
| 17α-Ethinylestradiol | estrogen |
| Estrone | estrogen |
| 4-Methoxy-Estrone | estrogen |
| 2-Methoxy-Estrone | estrogen |
| 17α-Hydroxypregnenolone | Progestogen |
| 17a-Hydroxyprogesterone | Progestogen |
| Progesterone | Progestogen |
| Pregnenolone | Progestogen |
| Pregnanediol | Progestogen |
| 5α-Pregnane-3,20-dione | Progestogen |
| 7-Ketocholesterol | sterols |
| Desmosterol | sterols |
| β-Sitosterol | sterols |
| 24,25-Dihydrolanosterol | sterols |
| 7α,25-Dihydroxycholesterol | sterols |
| 7a,27-Dihydroxycholesterol | sterols |
| 25-Hydroxycholesterol | sterols |
| 24-Hydroxycholesterol | sterols |
| 20α-Hydroxycholesterol | sterols |
| 7-Hydroxy-cholesten-3-one | sterols |
| Lathosterol | sterols |
| Cholesterol | sterols |
| Vitamin-D3 | Vitamin |

The table showed the 43 types of steroid hormones and their class we detected.
